# Multisite Quality Improvement Initiative to Identify and Address Racial Disparities and Deficiencies in Delivering Equitable, Patient-Centered Care for Multiple Myeloma—Exploring the Differences between Academic and Community Oncology Centers

**DOI:** 10.3390/curroncol30020123

**Published:** 2023-01-25

**Authors:** Joseph R. Mikhael, Shelby L. Sullivan, Jeffrey D. Carter, Cherilyn L. Heggen, Lindsay M. Gurska

**Affiliations:** 1 Translational Genomics Research Institute (TGen), City of Hope Comprehensive Cancer Center, Phoenix, AZ 85004, USA; 2 PRIME Education, LLC, Fort Lauderdale, FL 33309, USA

**Keywords:** quality improvement, racial disparities, multiple myeloma, barriers, community, academic

## Abstract

Treatment of multiple myeloma (MM) is complex; however, with equal access to care, clinical outcomes for Black patients match those in other patient groups. To reveal and begin to address clinical practice barriers to equitable, patient-centered MM care, this quality improvement (QI) initiative assessed patient electronic medical records (EMRs) and surveyed patients and providers at two large hospital systems and four community-based practices. For the educational intervention, providers participated in feedback-focused grand rounds sessions to reflect on system barriers and develop action plans to improve MM care. EMR reviews revealed infrequent documentation of cytogenetics and disease staging at community-based practices compared to large hospital systems. In surveys, providers from each care setting reported different challenges in MM care. Notably, the goals of treatment for patients and providers aligned at community clinics while providers and patients from large hospital systems had discordant perspectives. However, providers in community settings underreported race-associated barriers to care and identified different factors impacting treatment decision-making than Black patients. Relative to pre-session responses, providers were more likely to report high confidence after the educational sessions in aligning treatment decisions with guidelines and clinical evidence and shared decision-making (SDM). This QI study identified discordant perceptions among providers at large hospital systems and community-based practices in providing quality MM care. Provider education yielded increased confidence in and commitment to patient-centered care.

## 1. Introduction

Treatment decisions for multiple myeloma (MM) are complex, and a full understanding of disparities requires an analysis of factors across the patient journey from evaluation and risk assessment through shared decision-making (SDM) and patient-centered care. Notably, MM is the most common hematologic malignancy in Black/African American patients who experience a two-fold increase in lifetime risk and earlier age of onset compared with non-Black patients [1]. Relative to other groups, Black patients are less likely to receive or be referred to novel therapies, undergo transplants, and/or enroll in clinical trials [2,3,4,5,6,7,8,9,10,11,12]. However, with equitable access to care, clinical outcomes for Black patients match those observed in other patient groups, with some data suggesting a survival advantage [5,6,13,14]. In the absence of a clear biologic rationale for different MM treatment patterns, it is necessary to examine structural and other barriers to optimal care.

Of note, the underlying causes of racial disparities in MM care are multifactorial, with nonmedical barriers, including variable access to MM specialists due to geographic location [15,16], emerging as significant barriers to receiving equitable care. Evidence supports that MM specialists in large academic settings may rely more on risk stratification and guidelines informed by the latest clinical evidence to guide treatment decision-making than community oncologists [17]. These differences underly larger systemic discordances in care between academic and community settings, as, historically, academic centers are focused on specialty medical care and biomedical research, thereby having more access to clinical trials [18,19,20,21]. These differences are amplified when focused on the new era of precision medicine in cancer care, as academic centers typically have the expertise, facilities, support, and infrastructure to perform molecular testing techniques [18,22,23]. To provide quality patient care, academic and community centers have formed an alliance to ensure patients who receive care at community centers can still receive access to the molecular testing necessary to inform treatment decision-making [18,24]. However, despite the emerging adoption of this multidisciplinary partnership in cancer care, limited studies have performed a direct comparison to investigate whether racial disparities and deficiencies in care occur due to differences between receiving care at large academic versus community systems.

To determine whether a quality improvement (QI) initiative could reveal and begin to address clinical practice barriers to equitable, patient-centered MM care and whether these deficiencies differ between academic and community systems, electronic medical records (EMRs) and patient-provider tethered surveys were collected from two large academic systems and four community-based oncology practices. For the educational intervention, providers from the large hospital systems and community-based practices participated in feedback-focused grand rounds sessions to reflect on system barriers and develop action plans to improve MM care. Building on the design of prior QI programs [25,26,27,28,29], this QI study identified discordant perceptions between providers at large hospital systems and community-based practices in aligning their clinical practice with patient perspectives. Additionally, this study uncovered racial disparities that Black patients face in receiving MM care. Provider education yielded increased confidence in and commitment to providing patient-centered care.

## 2. Materials and Methods

**Figure 1:**
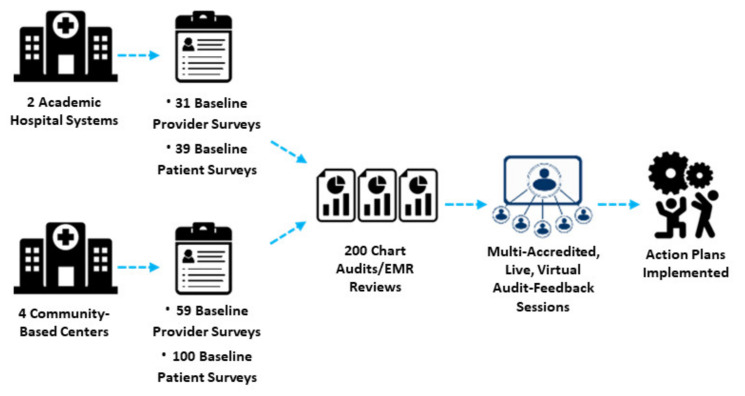
Schematic of Quality Improvement Methodology.

Beginning in April 2020, this QI study was conducted at two large hospital systems with the following scope: (a) a retrospective electronic medical record (EMR) audit to establish baseline care practices, (b) patient and provider surveys to assess beliefs and experiences with MM care, (c) a feedback-focused educational intervention to prompt team-based action plans, with Plan-Do-Study-Act (PDSA) implementation support, and (d) a follow-up EMR audit to assess documented changes in practice behavior (Figure 1). Beginning in August 2021, a scale-up QI study with similar program components was performed at four community-based practices. This program was part of a quality improvement initiative exempt from IRB oversight.

The criteria for inclusion in the EMR review included patients aged ≥ 18 years with a confirmed diagnosis of relapsed/refractory MM and at least two clinic visits within the prior 12 months. Patient charts were selected for audit by identifying patients meeting the inclusion criteria and most recently seen from the enrollment index. Working backward in time from the index date, charts were audited until the 200-patient chart cohort was generated. Chart variables included patient and disease characteristics, treatment history, clinical practice metrics, and patient-centered measures, including SDM. Patients and their providers completed 35-item and 30-item surveys, respectively (Supplemental Files S1–S4). Surveys included questions validated to assess racial differences among oncology patients in adherence, cancer beliefs, patient-provider communication, and awareness of health disparities [30,31,32,33,34,35]. *p*-values were calculated using Fisher’s exact *t*-test or Pearson’s chi-square test as indicated in Section 3.

In September 2020, providers at the two academic centers participated in a 1 h live feedback-focused educational session scheduled as part of each system’s grand rounds series to (a) critically assess their own MM practice patterns relative to national benchmarks, (b) prioritize areas for practice improvement, and (c) develop team action plans to implement over the following 6 months. From August to September 2021, providers at the four community-based practices also participated in 1 h live feedback-focused educational sessions. In all sessions, providers completed questionnaires before and after the sessions to evaluate changes in beliefs and confidence in MM care delivery. Systems receive ongoing PDSA support in the form of email and phone communications to assess progress in action-plan implementation and to identify residual needs.

## 3. Results

The analysis includes patient EMR data from two large hospital systems (*n*  =  200) and four community-based practices (*n* = 200); survey responses from patients (academic *n* = 39; community *n* = 100) and their providers (academic *n*  =  31; community *n* = 59); and among grand rounds participants (academic *n*  =  58; community *n* = 59), responses to pre- and post-session questionnaires and a qualitative assessment of their action plans (Table 1).

Baseline disease characteristics were disproportionally documented in EMRs, with community-based systems reporting fewer disease baseline characteristics than large hospital systems: disease stage (29% vs. 48%), cytogenetics (8% vs. 40%), and imaging (79% vs. 97%) (Table 2). While the documentation for patient-centered care practices was poorly documented at both academic systems and community clinics, patients at community clinics were more likely to have documented alcohol assessment (91% vs. 68%), advance care planning (70% vs. 25%), and involvement of an interprofessional team in diagnostic and prognostic tests (47% vs. 24%) compared to patients at academic systems; however, referral documentation was markedly lower at community clinics (5% vs. 20%) (Table 2). Most SDM practices were also infrequently documented at both large hospital systems and community clinics (Table 2).

In baseline surveys, providers from large hospital systems reported confidence in their treatment plan (22% vs. 18%) and patient health literacy (58% vs. 16%) as major challenges in MM care, while providers at community-based practices indicated affordability (32% vs. 7%) and individualizing treatment plans (41% vs. 12%) (Supplemental Table S1). Patients at community-based practices found confidence in their treatment plan as the biggest challenge in their MM care (32%); however, patients at large hospital systems revealed difficulty communicating with their care team about their concerns as their major challenge (25%) (Table 3). The top goal of community-based providers and patients in MM treatment aligned, with 47% of patients and 63% of providers selecting surviving as long as possible; however, discordance amongst providers and patients at large hospital systems was present, as 49% of patients selected controlling symptoms and improving quality of life as their top goals, and 56% of providers selected surviving as long as possible (Table 3). Providers at both large hospital and community-based settings reported discussing clinical trial enrollment with their patients (93% and 88%) and referring patients for enrollment (89% and 87%) (Table 3). By comparison, few patients recalled being asked about their interest in clinical trials (academic 15%; community 21%), being referred to a trial (academic 15%; community 17%), and/or enrollment in a trial (academic 15%; community 15%) (Table 3). Overall, patients and providers at community-based practices reported similar results in discussing the need for regular follow-up care and monitoring (83% vs. 79%) and the pros and cons of different treatment options (88% vs. 93%); however, responses from patients at large hospital settings did not match that of providers (67% vs. 85%; 65% vs. 81%) (Table 3). When asked about barriers to SDM, 28% of patients at large hospital systems reported not understanding what their care team is telling them and not knowing what to ask as the main barrier; while 47% of patients in community-based settings stated that they trust their care team to make the best decisions for them (Supplemental Table S1). Providers at both large hospital and community-based settings stated that not having enough time to engage in SDM as their biggest barrier (academic 50%; community 37%) (Supplemental Table S1).

Importantly, when questioned about barriers to care access for Black patients relative to other patients, providers at large hospital systems were more likely to report a problem for Black patients in having difficulty getting the best care because of their race/ethnic background (63% vs. 34%) than community providers (Table 3). Furthermore, 40% of patients treated at community clinics reported this as a barrier, suggesting that providers in community settings have discordant perspectives on racial disparities in MM care (Table 3). To explore this further, we investigated whether conflicting perspectives were reported in surveys from community providers and their patients based on race/ethnic background (Table 4). We observed that community providers (63%) and White patients (56%) both reported surviving as long as possible as their top goal for MM treatment, while Black/Latinx patients (46%) reported their top goal is improving their quality of life (Table 4). Furthermore, while 57% of community providers felt that risks, complications, and side effects associated with treatment were the most important factor for their patients in treatment decision-making, the majority of White patients (56%) reported treatment efficacy, and the majority of Black/Latinx patients (60%) reported effects on quality of life (Table 4). Interestingly, the majority of White patients (54%) also felt they were completely involved in their treatment decision-making, while 64% of Black/Latinx patients stated they were not involved in treatment decision-making because they trusted their care team to make the best decisions for them (Table 4). This coincides with 34% of Black/Latinx patients reporting that their care team could improve on providing education about MM treatment options (Table 4).

**Figure 2:**
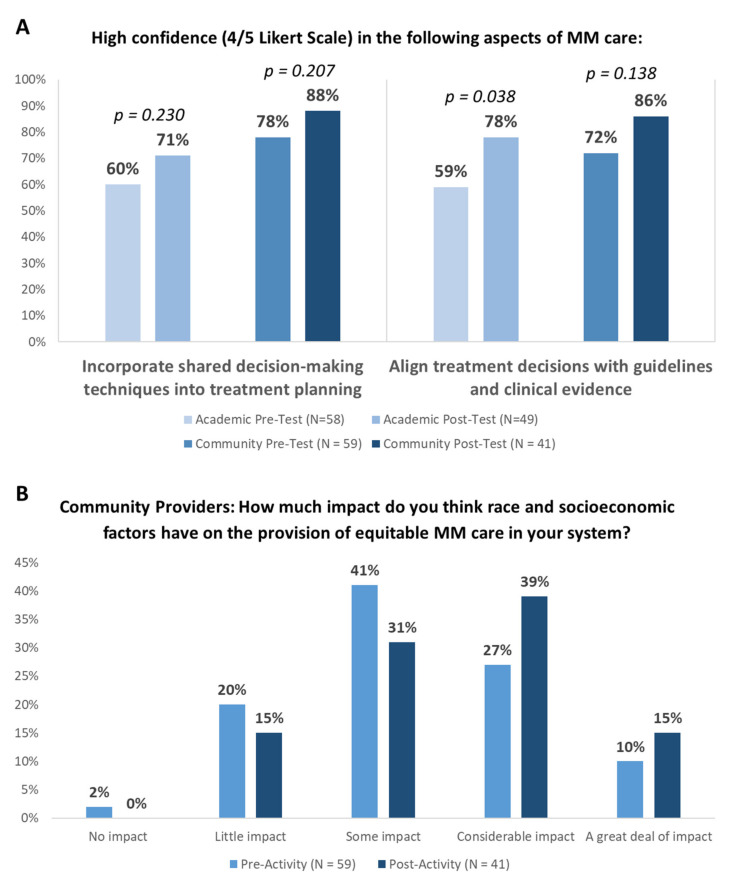
Educational Impact of Audit Feedback Sessions: (**A**) Proportion of providers reporting high confidence before and after the educational sessions. “High confidence” describes a score of 4–5 on a 5-point Likert scale (0, no confidence; 5, very high confidence). (**B**) Proportion of community providers reporting belief regarding the impact of race on equitable MM care. Fisher’s exact t-test was used.

Following the educational sessions, providers were significantly more likely to report high confidence to align treatment decisions with guidelines and clinical evidence (Figure 2A). Teams from academic systems prioritized addressing the following challenges: individualize treatment decision-making based on patient- and disease-related factors (37%), engaging patients in SDM (29%), and ensuring equitable access to novel therapies for all patients (24%) (Table 5). To accomplish this, teams from academic centers plan to identify patients at risk of relapse or refractory disease using prognostic scores, integrate a peer-reviewed template into the EMR to improve documentation, align treatment choice with guidelines and evidence, educate patients on new options, and encourage SDM. Similarly, teams from community centers prioritized addressing the following challenges: ensuring equitable access to novel therapies for all patients (29%), individualizing treatment decision-making based on patient- and disease-related factors (25%), engaging patients in SDM (17%), and providing adequate patient education about treatment options and potential side effects (17%) (Table 5). To accomplish this, teams from community centers plan to incorporate strategies to eliminate disparities in care for diverse MM patient populations, develop personalized treatment plans incorporating patient-specific factors and preferences, and enhance clinical trial discussion and enrollment. Importantly, following the educational sessions, community providers were more likely to recognize the impact of race and socioeconomic factors on the provision of equitable MM care in their systems (Figure 2B).

## 4. Discussion

We identified multiple potential signals of inequitable care that warrant further exploration, including the belief among 30% of patients treated at academic centers and 40% of patients treated at community clinics that they have difficulty getting the best care because of their race. Importantly, the majority of providers in our academic system cohort—but fewer providers in our community center cohort—identified patient race as a barrier to MM care. One hypothesis is that community clinics may be more accessible to Black patients, as supported by the underrepresentation of Black patients in MM clinical trials primarily underway at large hospital systems [4]. However, community providers have also been reported to underutilize MM treatment guidelines in treatment decision-making [17]. Alternatively, patients at community centers may be more aware of racial barriers, supported by the fact that 75% of these patients reported a problem for Black patients to afford the cost of health insurance and needed medical care compared to 12% of patients at large hospital systems, who may be unaware that their access to MM therapies is limited by entrenched barriers, including assumptions about race and treatment affordability.

While racial disparities in MM care are likely multifactorial, our QI program designed to identify barriers to equitable MM care at both large academic systems and community oncology practices uncovered unique differences in delivering MM care based on the type of practice. These include disproportional documentation in EMRs at community-based systems, which may be due to systemic barriers to obtaining disease staging, cytogenetic, and imaging data at community oncology clinics. Furthermore, there were marked differences in top challenges identified and goals of treatment between academic and community providers, as well as better alignment between community providers and patients in goals of treatment and reported discussion on regular follow-up and different treatment options compared to academic providers and patients. However, while alignment among community providers and patients prevailed, a deeper dive into racial disparities revealed that this alignment was restricted to community providers and White patients, with Black patients identifying different goals and important factors in treatment decision-making than their providers. The goal of care and treatment preferences identified through this study align with those found in prior studies of patients with MM and underscore the need for the engagement of patients in SDM to align treatment plans accordingly [36,37]. These findings correspond to the complexity of racial disparities in MM care and warrant further investigation to identify actionable plans for aligning provider and patient perspectives, regardless of race.

The National Comprehensive Cancer Network recommends clinical trial participation as the best option for MM management, yet Black patients are consistently underrepresented in oncology research [13]. Providers at both large hospital systems and community clinics reported frequent discussions about MM clinical trials, as well as high rates of referral for enrollment, yet few patients recalled these experiences. This discrepancy highlights the need for better patient education tools to support research participation and close enrollment gaps. Additional indicators of poor communication, such as providers misjudging the relative burden of their patients’ concerns about aspects of MM treatment, underscore the challenges of reaching a shared understanding. However, it is important to recognize that collaborative care between academic and community centers is also a critical element in increasing Black patient accrual in MM clinical trials, as the community healthcare system patient population represents greater racial, ethnic, and economic diversity, yet is often unaware of and/or has low participation in clinical trials [18,38]. An alliance between these types of healthcare systems has previously been shown to expand clinical trial diversity portfolios [18,39,40,41].

The provision of patient-centered care, including engagement in SDM, is a necessary component of efforts to overcome racial disparities in MM [42]. In this two-part QI study, we found low levels of chart-documented patient-centered care and SDM for all patients, irrespective of race; as well as provider- and patient-reported barriers to engaging in SDM. As an educational intervention, reflecting on system-based MM care practices is an effective method for increasing provider confidence in patient-centered care and SDM and facilitating plans for increased patient inclusivity. Follow-up EMR audits will measure whether these outcomes influence documented MM care.

To date, limited studies have performed a direct comparison to investigate whether racial disparities and deficiencies in care occur due to differences between receiving care at large academic systems versus community oncology clinics. In this study, we identified discordant perspectives between MM providers at academic systems and community clinics, as well as their patients. While racial disparities are prominent in our community clinic surveys, one caveat to this study is the lack of White patients participating in the patient surveys at academic systems, limiting us from comparing racial disparities between academic systems and community clinics. Looking ahead, with top discordances revealed in this study, future studies are better aligned to pinpoint whether racial disparities are the underlying causes of conflicting provider and patient perspectives. However, our comparison of deficiencies in care between academic systems and community clinics still requires attention to ensure that MM patients are receiving evidence-based-driven treatment and are engaged in their MM care journey.

Despite the innovative methodology and interesting results, several limitations were identified in the execution of this study. Most notably, the small sample sizes in both patient and provider survey numbers and patient charts collected from both academic and community sites limit the significance of conclusions that can be drawn from the data. However, the small group audit and feedback sessions allow for candid discussions of challenges, barriers, and potential solutions with well-constructed action plans for implementation. Additionally, the variation in self-reported race/ethnicity among patients participating in the study limits the comparability of results between groups, hampering the magnitude of racial inequalities uncovered throughout the study. Substantial differences in self-reported provider roles could introduce bias and potentially skew results, impeding conclusions. Future studies with similar methodology and larger sample sizes could help to reveal more significant gaps related to racial disparities and deficiencies in delivering equitable, patient-centered care for patients with MM. Furthermore, increasing awareness of discordances among academic and community providers and their patients with MM can elicit positive changes in a multidisciplinary approach to engaging patients in their care, aligning goals and preferences with treatments, and delivering the highest evidence-based, guideline-recommended care [43,44,45].

## 5. Conclusions

This QI study identified discordant perceptions among providers at large hospital systems and community-based practices in providing quality MM care. Specifically, while providers at community-based practices were more likely to align treatment goals with their patients than those at large hospital systems, community providers were more likely to underreport barriers to care due to a patient’s race and identified different factors impacting treatment decision-making than those of Black patients. Relative to pre-education session responses, providers were more likely to report high confidence after the educational sessions in aligning treatment decisions with guidelines and clinical evidence and SDM. The identified gaps can inform the delivery of future quality improvement programs to ensure the delivery of equitable care to MM patients.

## Figures and Tables

**Table 1:** Participant Demographics.

**Baseline Chart Audits**			
	**Academic Patients** (***n* = 200**)	**Community Patients** (***n* = 200**)	***p*-Value ^1^**
African American/Black	105 (53%)	32 (16%)	<0.001
Non-African American	95 (47%)	133 (67%)	
Race Not Documented	0 (0%)	35 (17%)	
Mean age	70 years	71 years	0.285
Male sex	68%	46%	0.001
**Patient and Provider Surveys**			
	**Academic Patients** (***n* = 39**)	**Community Patients** (***n* = 100**)	
Mean age	63 years	62 years	0.676
African American/Black	67%	47%	0.001
Hispanic/Latino	18%	3%	
Asian/Pacific Islander	10%	0%	
Native American/Alaska Native	5%	0%	
White	0%	50%	
Mean years attending clinic for MM care	3.5 years	5.5 years	0.032
	**Academic Providers ** (***n* = 31**)	**Community Providers** (***n* = 59**)	
Hematologist/Oncologist	65%	29%	0.011
Primary Care Physician/Other Physician	0%	14%	
NP/PA	12%	22%	
Nurse	23%	32%	
Other	0%	3%	
African American/Black	45%	8%	0.001
Caucasian/White	29%	75%	
Hispanic/Latino	16%	0%	
Asian/Pacific Islander	10%	17%	
Mean patients with MM seen per month	9 patients	49 patients	0.001
Mean years caring for patients with MM	17 years	9 years	0.001
**Feedback-Focused Educational Sessions**			
	**Academic Providers ** (***n* = 58**)	**Community Providers ** (***n* = 59**)	
Hematologist/Oncologist	84%	29%	0.001
NP/PA	5%	22%	
Nurse/Patient navigator/Case manager	10%	31%	
Primary Care Physician	0%	14%	
Other	0%	5%	
Learners’ self-reported monthly caseload of patients with MM (mean)	514 patients	1699 patients	0.001

^1^*p*-values were obtained from Pearson’s chi-square tests for categorical data. *p*-values were obtained from *t*-tests for means.

**Table 2:** Chart Documentation.

	Academic Patients (*n* = 200)	Community Patients (*n* = 200)	*p*-Value ^2^
**Disease Characteristics**			
Disease Stage	48%	29%	<0.001
Stage 1	24%	8%	<0.001
Stage 2	20%	8%	<0.001
Stage 3	4%	14%	<0.001
Stage Not Documented	52%	71%	<0.001
Cytogenetics	40%	8%	<0.001
t(4;14)	6%	4%	0.359
Imaging	97%	79%	<0.001
**Patient-Centered Care Practices**			
Referrals/ongoing management of comorbidities	20%	5%	<0.001
Response evaluation	85%	84%	0.777
Provision of patient education	24%	22%	0.634
Involvement of interprofessional team in diagnostic and prognostic tests	24%	47%	<0.001
Advance care planning documentation	25%	70%	<0.001
Assessment of side effects	25%	27%	0.648
Assessment of adherence	30%	37%	0.138
Assessment of patient-reported function	38%	49%	0.026
Assessment of patient-reported quality of life	39%	45%	0.224
Assessment of alcohol	68%	91%	<0.001
**Shared Decision-Making Practices**			
Checking for the patient/caregiver understanding of treatment options	24%	26%	0.644
Exploring patient/caregiver expectations for treatment outcomes	27%	26%	0.821
Ask patient or caregiver about treatment goals	28%	30%	0.659
Exploring patient/caregiver concerns and fears	31%	29%	0.663
Explaining pros and cons of treatment options	34%	41%	0.148
Providing treatment options	35%	37%	0.677
Providing opportunities for patient or caregiver to ask questions	52%	35%	<0.001

^2^*p*-values were obtained using Pearson’s chi-square tests.

**Table 3:** Patient and Provider Tethered Survey Findings.

	Academic			Community		
	**Providers** **(*n* = 31)**	**Patients** **(*n* = 39)**	***p*-value ^3^**	**Providers (*n* = 59)**	**Patients** **(*n* = 100)**	***p*-Value ^3^**
	**Challenges for Patients with MM**					
	Patient prompt: What is the biggest challenge you have faced in your MM care? Provider prompt: What is the biggest challenge your patients have faced in their MM care?					
Feeling confident in the treatment plan	22%	10%	0.196 *	18%	32%	0.067
Choosing whether to have/worry about complications of SCT	11%	0%	0.082 *	16%	7%	0.095
Lack of reliable transportation to and from care center	19%	20%	0.920	5%	10%	0.375 *
Worry about working or meeting responsibilities at home	22%	20%	0.841	18%	18%	0.920
Difficulty communicating with care team about concerns	15%	25%	0.335	5%	5%	>0.999
Worry about not having family/caregivers who can help	4%	18%	0.069 *	5%	14%	0.079
Worry about the cost of treatment/financial concerns	7%	7%	>0.999	32%	9%	<0.001
	**Goals of Treatment**					
	*Patient prompt:* What are your top 2 goals for MM treatment? *Provider prompt:* What do you believe are your patients’ top 2 goals for MM treatment?					
Controlling symptoms	26%	49%	0.050	34%	24%	0.177
Improving quality of life	52%	49%	0.807	59%	37%	0.006
Surviving as long as possible	56%	33%	0.071	63%	47%	0.055
Preventing progression or recurrence	22%	18%	0.623	25%	43%	0.026
Maintaining independence in daily activities	33%	15%	0.095	16%	16%	0.888
Staying out of the emergency room/hospital	11%	15%	0.721 *	4%	12%	0.032
Avoiding the need for a stem cell transplant	0%	5%	0.499 *	0%	3%	0.295 *
	**Treatment Decision-Making**					
	*Patient prompt:* Which of the following factors are the most important for your treatment-decision making? *Provider prompt:* Which of the following factors are the most important for your patients’ treatment decision-making?					
How well it will work against my/their cancer	56%	39%	0.171	50%	57%	0.450
Effects on quality of life	56%	44%	0.351	54%	45%	0.260
Risks/complications/side effects associated with the treatment	37%	46%	0.532	57%	29%	<0.001
Cost of treatment	19%	26%	0.532	21%	18%	0.718
Advice from loved ones	19%	31%	0.277	0%	13%	0.005 *
Advice/education from treatment team members	7%	13%	0.452 *	11%	22%	0.110
	**Clinical Trial Experience**					
	*Patient prompt:* Please describe your experience with clinical trials for MM (select all that apply). *Provider prompt:* Please rate how often you and your team do the following.					
*Patient prompt:* My doctor has asked about my interest in clinical trials *Provider prompt:* Discuss the possibility of clinical trial enrollment	93%	15%	<0.001	88%	21%	<0.001
*Patient prompt:* My doctor has referred me to a clinical trial *Provider prompt:* Refer patients for clinical trial enrollment	89%	15%	<0.001	87%	17%	<0.001
*Patient prompt:* I enrolled in a clinical trial	--	15%	--	--	15%	--
*Patient prompt:* No experience with clinical trials	--	59%	--	--	47%	--
	**Topics of Discussion**					
	*Patient prompt:* Please describe whether and how much your MM care team discussed the following topics with you. *Provider prompt:* Please rate how often you and your team discuss the following with your patients.					
Long-term side effects of cancer treatment for MM	85%	77%	0.474	96%	83%	0.011
The need for regular follow-up care and monitoring after completing treatment for MM	85%	67%	0.102	79%	83%	0.597
The pros and cons of different treatment options for MM	81%	65%	0.128	93%	88%	0.290
Patient’s goals and preference for treatment	81%	70%	0.277	100%	84%	0.001
	**Barriers to Care Access**					
	*Patient prompt:* Thinking about people like yourself, how much of a problem are these issues? *Provider prompt:* Please rate the degree of each problem (if present) for African American/Black patients relative to other patients.					
	**Having enough MM doctors or treatment centers near where patients live**					
Problem (any degree)	56%	34%	0.071	49%	61%	0.145
Major problem	7%	13%	0.452 *	15%	42%	<0.001
Minor problem	49%	21%	0.014	34%	19%	0.035
Not a problem	11%	10%	>0.999	34%	17%	0.015
Don’t know	33%	56%	0.044	18%	23%	0.517
	**Having difficulty getting the best care because of their race/ethnic background**					
Problem (any degree)	63%	30%	0.005	44%	40%	0.617
Major problem	30%	10%	0.277	13%	20%	0.303
Minor problem	33%	20%	0.264	21%	20%	>0.999
Not a problem	15%	15%	>0.999	47%	29%	0.019
Don’t know	22%	55%	0.004	19%	32%	0.067
	**Being able to afford the cost of health insurance and needed medical care**					
Problem (any degree)	86%	12%	<0.001	75%	75%	>0.999
Major problem	41%	5%	<0.001	31%	44%	0.092
Minor problem	45%	7%	<0.001	44%	31%	0.097
Not a problem	7%	10%	0.687 *	7%	12%	0.290
Don’t know	7%	78%	<0.001	19%	14%	0.439

^3^ *p*-values were obtained using Pearson’s chi-square tests unless marked with an asterisk (*) to indicate that the *p*-values were obtained using Fisher’s exact *t*-tests.

**Table 4:** Community Patient and Provider Survey Findings by Patient Race.

	Community Providers (*n* = 59)	Black/Latinx Patients (*n* = 50)	White Patients (*n* = 50)
**Goals of Treatment**			
*Patient prompt:* What are your top 2 goals for MM treatment? *Provider prompt:* What do you believe are your patients’ top 2 goals for MM treatment?			
Controlling symptoms	34%	26%	22%
Improving quality of life	59%	46%	28%
Surviving as long as possible	63%	38%	56%
Preventing progression or recurrence	25%	32%	54%
Maintaining independence in daily activities	16%	18%	14%
Staying out of the emergency room/hospital	4%	16%	8%
Avoiding the need for a stem cell transplant	0%	6%	4%
**Treatment Decision-Making**			
*Patient prompt:* Which of the following factors are the most important for your treatment decision-making? *Provider prompt:* Which of the following factors are the most important for your patients’ treatment decision-making?			
How well it will work against my/their cancer	50%	42%	56%
Effects on quality of life	54%	60%	42%
Risks/complications/side effects associated with the treatment	57%	36%	30%
Cost of treatment	21%	20%	16%
Advice from loved ones	0%	22%	8%
Advice/education from treatment team members	11%	10%	24%
**Shared Decision-Making (SDM)**			
*Patient prompt:* What keeps you from being more involved in treatment decision-making?			
I trust my care team to make the best decisions for me	--	64%	30%
I am too overwhelmed/worried to make a decision	--	28%	8%
I do not speak the same first language as my treating physician/treatment team members	--	18%	10%
I don’t know a lot about medicine or health, so I don’t really understand what my care team is telling me or I don’t know what to ask	--	14%	12%
My care team never asked what is important to me or what my goals of treatment are	--	14%	8%
I do not feel that my team values my opinions/listens to my concerns for my care	--	2%	6%
I feel that I am completely involved in my treatment decision making	--	14%	54%
**Areas of Improvement**			
*Patient prompt:* Which one aspect of your care do you think your MM care team could most improve?			
Education about MM and treatment options	--	34%	16%
Better provision of a translator/educational materials provided in my first language	--	26%	4%
Discussion about realistic treatment expectations and prognosis	--	28%	24%
Empathy throughout the emotional journey of managing my MM	--	32%	13%
Counseling to help me cope with my diagnosis and treatment	--	28%	16%
Insurance/financial counseling	--	14%	18%

**Table 5:** Audit Feedback Sessions.

Audit Feedback Sessions				
	Pre-Activity		Post-Activity	
	Academic Providers (*n* = 59)	Community Providers (*n* = 58)	Academic Providers (*n* = 41)	Community Providers (*n* = 49)
Pre-Activity: What is the biggest challenge to equitable MM care in your system? Post-Activity: Following this program, which challenge to equitable MM care do you intend to address with your team?				
Engaging patients in SDM	22%	17%	29%	38%
Individualizing treatment decision-making based on patient- and disease-related factors	16%	25%	37%	18%
Providing adequate patient education about treatment options and potential side effects	16%	17%	6%	28%
Integrating distress screening into patient monitoring	5%	12%	4%	13%
Ensuring equitable access to novel therapies for all patients	36%	29%	24%	3%
Other	5%	0%	0%	0%

## Data Availability

All new data obtained and analyzed in this study have been presented in this article.

## Supplementary Materials

The following supporting information can be downloaded at: https://www.mdpi.com/article/10.3390/curroncol30020123/s1, File S1. Academic Baseline Patient Survey Form; File S2. Academic Baseline Provider Survey Form; File S3. Community Baseline Patient Survey Form; File S4. Community Baseline Provider Survey Form; Table S1. Patient and Provider Survey Findings.
