# Supplementary material for: Multisite Quality Improvement Initiative to Identify and Address Racial Disparities and Deficiencies in Delivering Equitable, Patient-Centered Care for Multiple Myeloma—Exploring the Differences between Academic and Community Oncology Centers
Source: Curr Oncol. 2023 Jan 25;30(2):1598–613. doi: 10.3390/curroncol30020123 (PMC9955622; doi:10.3390/curroncol30020123)
Supplement: Supplementary file 1 [file curroncol-30-00123-s001.zip › Supplementary File 1_Academic Baseline Patient Survey Form.pdf]

____

PRIME®

PATIENT SURVEY

MULTIPLE MYELOMA HEALTH DISPARITIES

Thank you for taking this survey. Your participation in this survey is optional. Your responses will help health care professionals improve their care of people with multiple myeloma (MM). Please do not put your name on the survey. **Your answers will be confidential and anonymous.**

Information About You

1. What is your age in years? ____

2. What is your gender?

- ❑ **Male**
- ❑ **Female**

3. Which of the following best describes your race/ethnicity? Select 1

- ❑ African American/black
- ❑ Asian/Pacific Islander
- ❑ Native American/Alaska Native
- ❑ Caucasian/white
- ❑ Hispanic/Latino
- ❑ Other: ______________________________

4. What is the highest level of education you have completed? Select 1

- ❑ High school or GED
- ❑ Associate's degree
- ❑ Bachelor's degree
- ❑ Postgraduate (master's, PhD)
- ❑ Professional degree (MD, PharmD, DDS, JD)
- ❑ Other: ______________________________

5. What is your health insurance status? Select 1

- ❑ Commercial or private plan
- ❑ Federal-exchange insurance (Marketplace or Obamacare)
- ❑ Medicaid/Medicare
- ❑ No insurance

6. How confident are you filling out medical forms by yourself?

❍	❍	❍	❍	❍
**Not at all confident**	**Slightly confident**	**Moderately confident**	**Very confident**	**Extremely confident**

Information About Your Multiple Myeloma

7. How long have you been going to this clinic to receive care and treatment for your MM? (Give us your best estimate) ____ **years** ____ **months**

8. What are your top 2 goals for MM treatment? Select top 2

- ❑ Controlling symptoms
- ❑ Improving quality of life
- ❑ Surviving as long as possible
- ❑ Preventing progression or recurrence
- ❑ Maintaining independence in daily activities (eating, bathing, dressing)
- ❑ Staying out of the emergency room/hospital
- ❑ Avoiding the need for a stem cell transplant
- ❑ Other: ______________________________

9. Do you feel you are on track regarding your MM treatment goals? Select 1

- ❑ Yes, I am on track
- ❑ I feel somewhat on track
- ❑ No, I do not feel on track
- ❑ I am unsure; my care team does not give me progress updates
- ❑ I am unsure; I usually let my care team determine what treatment is best for me

10. Which of the following factors are the MOST important for your treatment decision-making? Select top 2

- ❑ How well it will work against my cancer
- ❑ Effects on quality of life
- ❑ Risks/complications/side effects associated with the treatment
- ❑ Cost of treatment
- ❑ Advice from loved ones
- ❑ Advice/education from treatment team members

CONTINUE ON NEXT PAGE

© 2020 PRIME Education, LLC. All Rights Reserved. www.primeinc.org

11. **What do you feel is the BIGGEST challenge that you have faced in your MM care?** Select 1

- ❑ Feeling confident that my treatment plan is the best plan for my cancer
- ❑ Choosing whether to have a stem cell transplant/ worry about the complications of transplant
- ❑ Lack of reliable transportation to and from my care center
- ❑ Worry about being unable to work or meet responsibilities at home
- ❑ Worry about not having family or other caregivers who can help me
- ❑ Difficulty communicating with my care team about my concerns
- ❑ Worry about the cost of treatment/financial concerns
- ❑ Other: ______________________________

This set of questions relates to your experience taking oral medications for MM. Please describe how often, if ever, you have:

12. **Forgotten to take your medication**

❍ Never ❍ Rarely ❍ Sometimes ❍ Often ❍ Always

13. **Skipped or stopped taking medicine because you didn't think it was working**

❍ Never ❍ Rarely ❍ Sometimes ❍ Often ❍ Always

14. **Skipped or stopped taking medicine because it made you feel bad**

❍ Never ❍ Rarely ❍ Sometimes ❍ Often ❍ Always

15. **Skipped, stopped, not refilled, or taken less medicine because of the cost**

❍ Never ❍ Rarely ❍ Sometimes ❍ Often ❍ Always

16. **Not had medicine with you when it was time to take it**

❍ Never ❍ Rarely ❍ Sometimes ❍ Often ❍ Always

17. **Please describe your experience with clinical trials for MM.** select all that apply

- ❑ My doctor has asked me about my interest in clinical trials
- ❑ My doctor has referred me to a clinical trial
- ❑ I enrolled in a clinical trial
- ❑ No experience with clinical trials

This set of questions explores general beliefs about cancer. Please rate how much you agree or disagree with the following statements.

18. **There's not much you can do to lower your chances of getting cancer.**

❍ Strongly disagree ❍ Disagree ❍ Neutral ❍ Agree ❍ Strongly Agree

19. **It seems like everything causes cancer.**

❍ Strongly disagree ❍ Disagree ❍ Neutral ❍ Agree ❍ Strongly Agree

20. **There are so many different recommendations about preventing cancer, it's hard to know which ones to follow.**

❍ Strongly disagree ❍ Disagree ❍ Neutral ❍ Agree ❍ Strongly Agree

21. **When I think about cancer, I automatically think about death.**

❍ Strongly disagree ❍ Disagree ❍ Neutral ❍ Agree ❍ Strongly Agree

Communication With Your Multiple Myeloma Care Team

Please describe whether and how much your MM care team discussed the following topics with you.

22. **Results of genetic testing on your type of MM**

❍ Did not discuss ❍ Briefly discussed ❍ Discussed in detail

23. **Your goals and preferences for treatment**

❍ Did not discuss ❍ Briefly discussed ❍ Discussed in detail

24. **The pros and cons of different treatment options for MM**

❍ Did not discuss ❍ Briefly discussed ❍ Discussed in detail

25. **The need for regular follow-up care and monitoring after completing treatment for MM**

❍ Did not discuss ❍ Briefly discussed ❍ Discussed in detail

CONTINUE ON NEXT PAGE

© 2020 PRIME Education, LLC. All Rights Reserved. www.primeinc.org

26. Long-term side effects of cancer treatment for MM

❍ Did not discuss ❍ Briefly discussed ❍ Discussed in detail

27. Emotional or social needs related to cancer

❍ Did not discuss ❍ Briefly discussed ❍ Discussed in detail

28. Lifestyle or health recommendations

❍ Did not discuss ❍ Briefly discussed ❍ Discussed in detail

29. Please select any of the following statements that are TRUE about your doctor. **Select all that apply**

- ❑ My doctor has expressed concerns about my feelings.
- ❑ My doctor respects me as a person.
- ❑ My doctor lets me say what is important to me.
- ❑ My doctor speaks quickly and uses complex words.
- ❑ My doctor asks me about my preferences for treatment.
- ❑ My doctor and I decide on my treatment plan together.
- ❑ My doctor treats me differently from other patients because of my race.

30. What type of education helps you to learn about your diagnosis and your treatment options the BEST? **Select top 2**

- ❑ Written handouts/pamphlets (in my first language)
- ❑ Video/visual-aid resources that I can access online or on my mobile device
- ❑ I prefer to learn with the help of my care team/in my provider's office before/during/after my appointment
- ❑ I prefer to learn in the privacy of my own home
- ❑ I prefer to learn in a group setting with the support of others going through the same thing
- ❑ Other: ______________________________

31. What keeps you from being more involved in treatment decision-making?

- ❑ I trust my care team to make the best decisions for me
- ❑ I am too overwhelmed/worried to make a decision
- ❑ I do not speak the same first language as my treating physician/treatment team members
- ❑ I don't know a lot about medicine or health, so I don't really understand what my care team is telling me/I don't know what to ask
- ❑ My care team never asked what is important to me or what my goals of treatment are
- ❑ I do not feel that my team values my opinions/listens to my concerns for my care
- ❑ Other: ______________________________
- ❑ I feel that I am completely involved in my treatment decision-making

This set of questions explores your experiences with access to care. Thinking about other people like yourself, how much of a problem are these?

32. Being able to afford the cost of health insurance and needed medical care

- ❑ Major problem
- ❑ Minor problem
- ❑ Not a problem at all
- ❑ Don't know

33. Having enough MM doctors or treatment centers near where they live

- ❑ Major problem
- ❑ Minor problem
- ❑ Not a problem at all
- ❑ Don't know

34. Having difficulty getting the best care because of their race or ethnic background

- ❑ Major problem
- ❑ Minor problem
- ❑ Not a problem at all
- ❑ Don't know

35. Which one aspect of your care do you think your MM care team could most improve?

- ❑ Education about MM and treatment options
- ❑ Better provision of a translator/educational materials provided in my first language
- ❑ Discussion about realistic treatment expectations and prognosis
- ❑ Empathy throughout the emotional journey of managing my MM
- ❑ Counseling to help me cope with my diagnosis and treatment
- ❑ Insurance/financial counseling
- ❑ Other: ______________________________

THANK YOU FOR TAKING THIS SURVEY

© 2020 PRIME Education, LLC. All Rights Reserved.
