# Supplementary material for: Multisite Quality Improvement Initiative to Identify and Address Racial Disparities and Deficiencies in Delivering Equitable, Patient-Centered Care for Multiple Myeloma—Exploring the Differences between Academic and Community Oncology Centers
Source: Curr Oncol. 2023 Jan 25;30(2):1598–613. doi: 10.3390/curroncol30020123 (PMC9955622; doi:10.3390/curroncol30020123)
Supplement: Supplementary file 1 [file curroncol-30-00123-s001.zip › Supplementary File 4_Community Baseline Provider Survey Form.pdf]

______

PRIME®

MM PROVIDER SURVEY

ADVANCING COMMUNITY-BASED MULTIPLE MYELOMA CARE

Thank you for completing this survey for the program in multiple myeloma (MM) in which you are participating. Your responses remain confidential and anonymous. Some of the survey questions ask you to estimate aspects of your patients' MM and treatment. Unless instructed otherwise, please estimate by considering the specific patients in your clinic who did (or will) complete the patient surveys for this program.

Information About You

1. What is your age? _________ years old

2. Please indicate your gender:

- ❑ Male
- ❑ Female
- ❑ Other: ________________________

3. Which of the following best describes your race/ethnicity? Select 1

- ❑ African American/Black
- ❑ Caucasian/White
- ❑ Hispanic/Latinx
- ❑ Asian/Pacific Islander
- ❑ Native American/Alaska Native
- ❑ Other: ________________________

4. What is your role within the interprofessional team?

- ❑ Hematologist/oncologist
- ❑ Physician assistant
- ❑ Nurse practitioner
- ❑ Nurse/nurse navigator
- ❑ Other: ________________________

5. How many years have you been caring for patients with MM? ______

6. Approximately how many patients with MM do you provide care for each month? ______

Patient Care for Multiple Myeloma

7. Which of the following patient/disease characteristics do you routinely document for patients with MM? Select all that apply

- ❑ Disease stage (eg, ISS stage)
- ❑ Cytogenetics (eg, *t*(4;14), *t*(11;14), TP53 mutations, 17p deletion, monosomy 17)
- ❑ Comorbidity burden (eg, Charleston comorbidity index)
- ❑ Myeloma-defining events (eg, hypercalcemia, renal insufficiency, anemia, bone lesions)
- ❑ Immunoglobulin levels (eg, IgG, IgA, IgM)

8. What is the MOST challenging issue you encounter in managing patients with MM? Select 1

- ❑ Individualizing treatment plans
- ❑ Knowing what my patients' treatment goals are
- ❑ Engaging patients in shared decision-making
- ❑ Patient health literacy
- ❑ Patient non-adherence/lack of follow-up
- ❑ Supportive care counseling (quality of life, etc.)

9. What do you believe are your patients' top 2 goals for MM treatment? Select top 2

- ❑ Controlling symptoms
- ❑ Improving quality of life
- ❑ Surviving as long as possible
- ❑ Preventing progression or recurrence
- ❑ Maintaining independence in daily activities (eating, bathing, dressing)
- ❑ Staying out of the emergency room/hospital
- ❑ Avoiding the need for a stem cell transplant
- ❑ Other: ________________________

CONTINUE ON NEXT PAGE

© 2021 PRIME Education, LLC. All Rights Reserved.

10. What percentage of your patients do you estimate are on track to achieve their treatment goals? [Estimate to the nearest 5%] ______

11. Which of the following factors are the MOST important to your patients in their treatment decision-making? Select top 2

- ❑ How well it will work against their cancer
- ❑ Effects on quality of life
- ❑ Risks/complications/side effects associated with the treatment
- ❑ Cost of treatment
- ❑ Advice from loved ones
- ❑ Advice/education from treatment team members

12. In addition to clinical guidelines, which factors do you consider MOST important for treatment decision-making for patients with MM? Select top 2

- ❑ Treatment effectiveness
- ❑ Effects on quality of life
- ❑ The patient's goals for treatment
- ❑ The patient's concerns for risks/complications/side effects
- ❑ Cost of treatment
- ❑ Likelihood of patient adherence
- ❑ Other: ________________________

13. What do you feel is the BIGGEST challenge your patients face in their MM care? Select 1

- ❑ Feeling confident that their treatment plan is the best plan for their cancer
- ❑ Choosing whether to have a stem cell transplant/ worry about the complications of transplant
- ❑ Lack of reliable transportation to and from their care center
- ❑ Worry about being unable to work or meet responsibilities at home
- ❑ Worry about not having family or other caregivers who can help them
- ❑ Difficulty communicating with their care team about their concerns
- ❑ Worry about the cost of treatment/financial concerns
- ❑ Other: ________________________

14. What percentage of your patients do you estimate are adherent to their oral medications for MM? [Estimate to the nearest 5%] ______

Communication with Your Patient

Please rate how often you and your team discuss the following with your patients:

15. Results of genetic testing

❍ Do not discuss ❍ Briefly discuss ❍ Dicuss in detail

16. Patients' goals and preferences for treatment

❍ Do not discuss ❍ Briefly discuss ❍ Dicuss in detail

17. The pros and cons of different treatment options for MM

❍ Do not discuss ❍ Briefly discuss ❍ Dicuss in detail

18. The need for regular follow-up care and monitoring after completing treatment for MM

❍ Do not discuss ❍ Briefly discuss ❍ Dicuss in detail

19. Long-term side effects of cancer treatment for MM

❍ Do not discuss ❍ Briefly discuss ❍ Dicuss in detail

20. Emotional or social needs related to cancer

❍ Do not discuss ❍ Briefly discuss ❍ Dicuss in detail

21. Lifestyle or health recommendations

❍ Do not discuss ❍ Briefly discuss ❍ Dicuss in detail

CONTINUE ON NEXT PAGE

© 2021 PRIME Education, LLC. All Rights Reserved.

Please rate how often you and your team perform the following:

22. Discuss the possibility of clinical trial enrollment

❍ Never ❍ Rarely ❍ Sometimes ❍ Often ❍ Always

23. Refer patients for clinical trial enrollment

❍ Never ❍ Rarely ❍ Sometimes ❍ Often ❍ Always

24. Express concerns about patients' feelings

❍ Never ❍ Rarely ❍ Sometimes ❍ Often ❍ Always

25. Ask patients about their MM treatment preferences

❍ Never ❍ Rarely ❍ Sometimes ❍ Often ❍ Always

Please rate the degree of the problem (if present) for African American patients with MM relative to patients from other racial/ethnic groups.

26. Being able to afford the cost of health insurance and needed medical care

❍ Major problem ❍ Minor problem ❍ Not a problem at all ❍ Don't know

27. Having enough MM doctors or treatment centers near where they live

❍ Major problem ❍ Minor problem ❍ Not a problem at all ❍ Don't know

28. Having difficulty getting the best care because of their race or ethnic background

❍ Major problem ❍ Minor problem ❍ Not a problem at all ❍ Don't know

29. What types of educational materials/resources do you provide to your patients? **Select all that apply**

- ❑ Written handouts/pamphlets (in patient's first language)
- ❑ Video/visual-aid resources that patients can access on their mobile devices
- ❑ One-on-one discussions with someone from your care team before, during, or after the patient's appointment
- ❑ Materials patients can use to learn in the privacy of their own home
- ❑ Opportunities for group discussions where patients can learn and get support from each other
- ❑ Other: ________________________

30. What is your BIGGEST barrier to engaging your patients with MM in shared decision-making (SDM)? **Select 1**

- ❑ Not enough time to engage in SDM
- ❑ Lack of confidence to engage in SDM
- ❑ Lack of staff to support SDM
- ❑ Patient resistance to SDM
- ❑ Patients' low health literacy
- ❑ Other: ________________________

THANK YOU FOR COMPLETING THIS SURVEY

© 2021 PRIME Education, LLC. All Rights Reserved.
