# Supplementary material for: Multisite Quality Improvement Initiative to Identify and Address Racial Disparities and Deficiencies in Delivering Equitable, Patient-Centered Care for Multiple Myeloma—Exploring the Differences between Academic and Community Oncology Centers
Source: Curr Oncol. 2023 Jan 25;30(2):1598–613. doi: 10.3390/curroncol30020123 (PMC9955622; doi:10.3390/curroncol30020123)
Supplement: Supplementary file 1 [file curroncol-30-00123-s001.zip › Supplementary Table S1_Patient and Provider Survey Findings.pdf]

Current Oncology

MDPI

Supplemental Table 1. Patient and Provider Survey Findings.

	Academic Providers (N = 31)	Community Providers (N = 59)	Academic Patients (N=39)	Community Patients (N=100)
Challenges for Patients with MM				
Provider prompt: What is the single most challenging issue you encounter in managing patients with MM?				
Patient health literacy	58%	16%	--	--
Engaging patients in shared-decision making	12%	9%	--	--
Individualizing treatment plans	12%	41%	--	--
Supportive care counseling	12%	5%	--	--
Patient non-adherence/lack of follow-up	3%	18%	--	--
Knowing what my patients' treatment goals are	3%	11%	--	--
**Shared Decision Making (SDM)**				
Patient prompt: What keeps you from being more involved in treatment decision making?				
I trust my care team to make the best decisions for me	--	--	23%	47%
I am too overwhelmed/worried to make a decision	--	--	5%	18%
I do not speak the same first language as my treating physician/treatment team members	--	--	15%	14%
I don't know a lot about medicine or health, so I don't really understand what my care team is telling me or I don't know what to ask	--	--	28%	13%
My care team never asked what is important to me or what my goals of treatment are	--	--	15%	11%
I do not feel that my team values my opinions/listens to my concerns for my care	--	--	10%	4%
I feel that I am completely involved in my treatment decision making	--	--	3%	34%
Provider prompt: What is the biggest barrier to engaging your patients with MM in shared decision making?				
Not enough time to engage in SDM	50%	37%	--	--
Lack of confidence to engage in SDM	15%	11%	--	--
Lack of staff to support SDM	39%	14%	--	--
Patient resistance to SDM	35%	11%	--	--
Patients' low health literacy	42%	25%	--	--
**Patient Experience with Patient-Provider Communication**				
Patient prompt: Please select any of the following statements that are true about your doctor.				
My doctor respects me as a person	--	--	59%	52%
My doctor lets me say what is important to me	--	--	46%	49%
My doctor has expressed concerns about my feelings	--	--	41%	32%
My doctor asks me about my preferences for treatment	--	--	39%	32%
My doctor and I decide on my treatment plan together	--	--	31%	52%
My doctor speaks quickly and uses complex words	--	--	56%	26%
My doctor treats me differently from other patients because of my race	--	--	21%	5%
**Areas of Improvement**				
Patient prompt: Which one aspect of your care do you think your MM care team could most improve?				
Education about MM and treatment options	--	--	16%	26%
Better provision of a translator/educational materials provided in my first language	--	--	11%	18%
Discussion about realistic treatment expectations and prognosis	--	--	21%	30%
Empathy throughout the emotional journey of	--	--	29%	26%

managing my MM				
Counseling to help me cope with my diagnosis and treatment	--	--	16%	24%
Insurance/financial counseling	--	--	8%	19%
